# Silicasomes in Oncology: From Conventional Chemotherapy to Combined Immunotherapy

**DOI:** 10.3390/molecules30061257

**Published:** 2025-03-11

**Authors:** Alicia Arroyo-Nogales, Guillermo Plaza-Palomo, Javier González-Larre, Sandra Jiménez-Falcao, Alejandro Baeza

**Affiliations:** Materials and Aerospace Production Department, Superior Technic School of Aeronautics and Space Engineering, Politechnic University of Madrid Department Materiales y Producción Aeroespacial, ETSI Aeronáutica y del Espacio, Universidad Politécnica de Madrid, 28040 Madrid, Spain; a.arroyon@alumnos.upm.es (A.A.-N.); guillermo.plaza@alumnos.upm.es (G.P.-P.); j.glarre@upm.es (J.G.-L.); sandra.jfalcao@upm.es (S.J.-F.)

**Keywords:** nanomedicine, nanooncology, silicasomes, protocells, drug delivery, controlled release

## Abstract

The use of nanoparticles as drug carriers in oncology has evolved from their traditional role as chemotherapy carriers to their application in immunotherapy, exploiting not only their passive accumulation in solid tumors but also their ability to interact with immune cells. Silicasomes are highly versatile nanoplatforms composed of a mesoporous silica core whose external surface is coated with a lipid bilayer that allows the co-delivery of therapeutic agents having different chemical natures (small molecules, proteins, enzymes, or oligonucleotides, among others). Herein, cutting-edge advances carried out in the development and application of silicasomes are presented, providing a general description of the performance of these nanotransporters. Additionally, the specific load of chemotherapeutic drugs is explored, followed by a discussion of the immunotherapeutic application of silicasomes and the combination of different therapeutic strategies, including theragnosis, in a single silicasome platform, highlighting the enormous potential of these nanosystems.

## 1. Introduction

During the last decades, different therapeutic strategies have emerged to defeat cancer, from the development of targeted nanomedicines based on smart nanomaterials [1] to cell-based therapies that employ genetically engineered immune cells to eradicate tumors with extreme precision and selectivity [2]. In the field of nanooncology, the starting point of this technology was the discovery of the passive accumulation of nanoparticles (NPs) within solid tumors carried out by the Japanese researchers Maeda and Matsumura, a phenomenon known as the *enhanced permeation and retention* (EPR) effect [3]. The accelerated growth of tumoral masses requires the rapid formation of tumoral blood vessels to provide nutrients to the new cells. Therefore, these tumoral vessels are sloppily constructed, presenting nanometric pores through which NPs can be extravasated (*enhanced permeation*). Additionally, the growth of tumoral mass induces the collapse of the lymphatic drainage system, which leads to an enhanced accumulation of the extravasated NPs (*enhanced retention*) [4]. The discovery of this effect provided hope for developing smart nanocarriers having the capacity to deliver chemotherapeutic agents to tumoral cells in a safe, selective, and efficient manner. Unfortunately, after more than 40 years of intense research, only a reduced number of nanomedicines have reached the clinic [5]. The reasons for this disappointing outcome are varied: difficulties associated with the production of the nanocarriers, lack of proper regulation for nanomedicines, and technical/biological barriers such as aggregation, immune clearance, and lack of tumor penetration [6], just to quote a few, which hamper the performance of these nanosystems in biological tissues [7].

**Figure 1:**
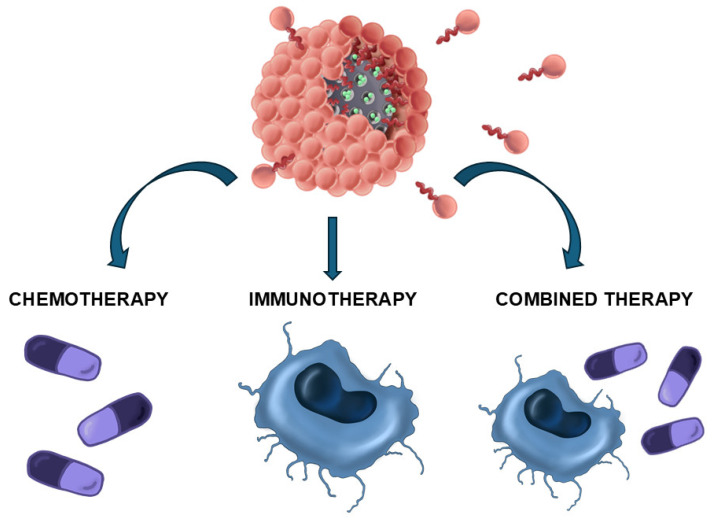
Schematic representation of the disassembly of silicasomes in order to deliver a variety of therapeutic agents.

Moreover, the EPR effect itself is the subject of intense criticism because it is not universal in human tumors and even presents strong heterogeneity between patients affected by the same tumor type. In a recent study, Chan et al. reported that only 0.7% (median) of administered NPs are accumulated in solid tumors [8]. Therefore, the conventional dogma of the suitability to employ nanometric carriers to deliver chemotherapy has been subject to intense debate [9]. In the last years, the nanooncology field has experienced a paradigm shift, from just targeting the tumoral cells to focusing attention on the cells of the immune system, with the objective to trigger a potent antitumoral response that will eradicate the tumor [10]. Throughout the time of their development, tumoral cells play an intricate dance with the immune system, known as the three E’s process (*Elimination–Equilibrium–Escape*) [11]. In the first stage, the immune cells are capable to detect and destroy the early tumoral cells (Elimination). After a certain period of time, the tumoral cells become partially stealthy towards the immune cells, which cannot fully eliminate the malignant cells, reaching a stationary stage (Equilibrium) that can last even years without symptomatology. Finally, the tumoral cells gain critical skills that allow them to overcome the immune system’s control and, in many cases, to hijack immune cells to support tumor progression (Escape). In recent years, a myriad of different nanomedicines have been engineered to revert the Escape phase through the delivery of immunomodulators or the generation of danger signals by hyperthermia, just to quote a few mechanisms, with the aim to restore the normal function of immune system against the tumor [12]. Concern about the release of drugs from these nanocarriers [13] has led to the development of core shell structures in which the load is protected by an outer shield. Many of these approaches described in the literature consist of a combination of organic shells encapsulating inorganic nanoparticles [14]. Herein, recent advances carried out in the development and application of one specific family of nanocarriers, named silicasomes or protocells, will be presented. A silicasome or protocell is a type of nanocarrier composed of a mesoporous silica core coated with a lipid bilayer that avoids the premature release of the housed drugs (Figure 1) [15,16]. Silicasomes present interesting properties as a result of their hybrid organo-inorganic nature. Thus, these nanocarriers present the following: (1) high loading capacity due to the presence of the porous silica network, (2) excellent colloidal stability, (3) almost zero premature release behavior due to the presence of the lipid shell, (4) high biocompatibility, and (5) an easy-to-tune nature due to the great versatility in pore architecture and lipid composition, which allow the incorporation of many different functional groups on their external surface. Additionally, the hybrid nature of silicasomes allows for the delivery of multiple therapeutic agents that exhibit different chemical natures, from hydrophilic drugs or macromolecules retained within the pore silica network to hydrophobic molecules that can be housed in the lipidic bilayer [17]. Due to these properties, silicasomes are not only excellent nanomaterials to deliver chemo- or immunotherapeutic agents but, perhaps more importantly, they are ideal for administering combinations of both in order to achieve synergic effects that lead to the complete tumor eradication.

There are few articles in the literature providing different approaches to the importance and potential of silicasomes: from generalistic reviews, which barely mention the possibility of a combination of silica nanoparticles with lipidic structures yielding silicasomes [18], to more technical and specialized perspectives evaluating synthetic, structural, or functional features of silicasomes [19,20]. The aim of this work is to gather the most recent advances in the field of silicasomes regarding the therapeutic motivation, whether it is a chemotherapeutic agent, immunotherapy, or a combined approach (including theragnosis), filling a gap in the literature that has not yet been covered, to the best of our knowledge.

## 2. Silicasomes as Chemotherapeutic Vehicles

Chemotherapy is regarded as one of the first-line treatments for cancer, along with surgery and radiotherapy. The mechanism of action of most chemotherapeutic drugs typically consists of the disruption of DNA replication or cell division machinery, limiting the speed at which cancerous cells replicate [21]. Although it is one of the most cost-effective ways to treat cancer, it leads to severe side effects associated with its high toxicity as a consequence of the lack of selectivity towards cancer cells [22]. Therefore, current strategies for chemotherapeutic approaches aim to reduce this unwanted toxicity derived from the therapy. In this context, the use of silicasomes envisions an optimistic future for chemotherapy, since silicasomes provide a variety of features that enable the release of well-known chemotherapeutic agents in a more efficient, selective, and safer manner.

One of the main drawbacks of traditional chemotherapy is a low internalization rate because of an inefficient reaching of the tumor cells. In contrast, the use of silicasomes permits the selective direction of the therapy to the site of action by the incorporation of targeting moieties through a variety of chemical strategies to the outer surface of the nanovehicles, increasing the rate of internalization of the NPs. This fact is possible thanks to the chemical modifications that can be carried out in the outer membrane of silicasomes, exhibiting the targeting moieties. Hence, the design of effective targeting moieties remains a challenge that could be a game-changer for cancer surveillance [23]. As an example, Villaverde et al. designed a synthetic analogue of norepinephrine, *p*-amino benzyl guanidine, which selectively interacts with the norepinephrine transporter, which is overexpressed in 95% of neuroblastoma cells [24]. In a subsequent work, Parra-Nieto et al. used this targeting element to successfully deliver doxorubicin (DOX) to neuroblastoma cells using silicasomes as nanovehicles [25].

**Figure 2:**
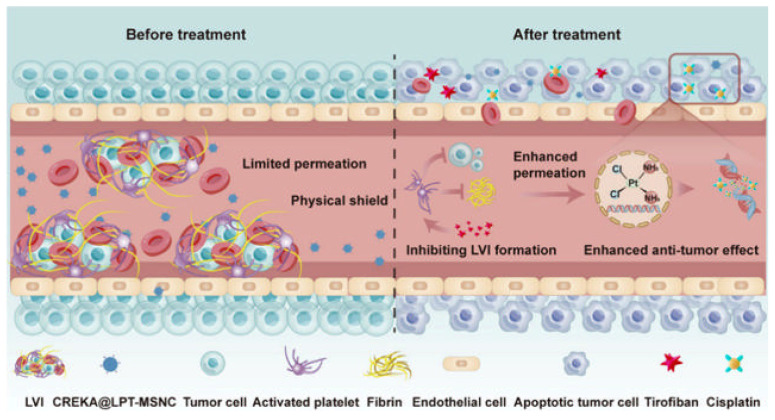
Schematic illustration of the CREKA modified, cisplatin-loaded silicasome (CREKA@LPT-MSNC) treatment to prevent LVI and improve the response of the tumor cells to the chemotherapeutic drug. Reprinted with permission from reference [28]. Copyright © 2024 American Chemical Society.

A common element that is usually anchored to the surface of silicasomes are PEG moieties. This polymer might be included into the nanotransporter’s architecture to achieve different functionalities like targeting or providing colloidal stability. As an example, Meng et al. designed PEI/PEG coated silicasomes for stromal targeting and gemcitabine and paclitaxel delivery to treat pancreatic cancer [26], while Liu et al. used PEG to improve colloidal stability and prolonged blood circulation time in a silicasome system loaded with a platinum drug [27]. Another example of surface functionalization permits the avoidance of platelet adhesion in the treatment of bladder cancer, as presented in a recent study, in which a cisplatin-loaded silicasome was modified with a fibrin-binding peptide (CREKA), consequently reducing lymphovascular invasion and therefore reducing the risk of metastasis and notably improving the tumor’s permeability to the cisplatin, without any observed adverse effects (Figure 2) [28].

A difficulty that must be overcome in traditional chemotherapy is to achieve a reduction in the dose administered to the patients and therefore reduce undesirable side effects. The low solubility of some drugs in biological media leads to the administration of large amounts of chemotherapies in order to reach a certain effective concentration. Fortunately, silicasomes also provide a solution for this challenge, since, because of their amphiphilic nature, they allow the transport of both hydrophilic (loaded within the silica core) and hydrophobic drugs (entrapped within the lipid bilayer), ameliorating their bioavailability. This characteristic is of special interest, because it permits not only the transport of a variety of different drugs but also their simultaneous delivery, optimizing the dose of each individual drug and minimizing their toxic effect. Hence, synergistic effects between both drugs can be achieved, reducing their individual dose while improving their therapeutic effect [26].

Despite other nanotransporters having been described in the literature that have high loading capacity, like mesoporous silica NPs, silicasomes offer a more advanced and safer solution for chemotherapy delivery, since the presence of the lipid bilayer avoids premature leakage of the load by sealing the pores of the silica until it reaches the site of delivery. In addition, the presence of the lipid bilayer also protects the silica from early degradation by hydrolysis. Once in the targeted site of action, and with proper design of the nanotransporter, the release of the drug can be controlled by different stimuli, whether exogenous, endogenous, or a combination of both [29]. They designed a sophisticated nanosystem controlled by three different stimuli (reduced glutathione, pH, and light irradiation) and constituted by a liposome loaded with DOX and cerium NPs for breast cancer treatment [30].

**Figure 3:**
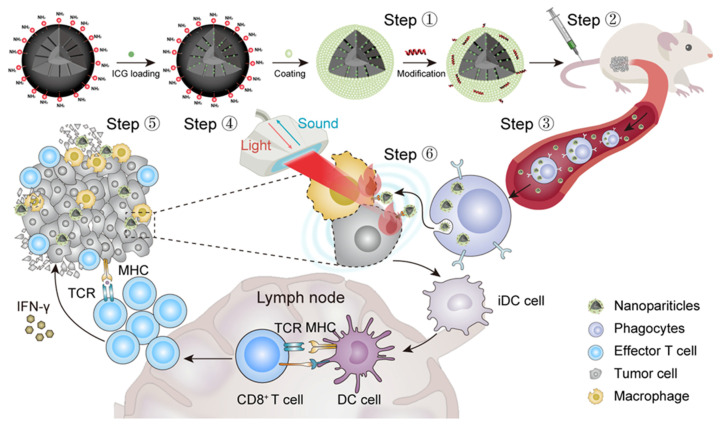
Schematic representation of the strategy followed by Wang et al. to improve the penetration of silicasomes in a solid tumor based on the incorporation of an ApoA-1 mimetic peptide on the surface of the nanotransporter. Reprinted with permission from reference [31]. Copyright © 2023 Elsevier.

Regarding the limitations that nanooncology faces in the treatment of solid tumors, one of the main barriers that must be considered is the presence of high stromal density due to fibrotic tissue formation. This hinders the penetration of the nanocarriers into the deeper layers of the tumor, not only because of the presence of a physical barrier but also because of the increased interstitial pressure existing in the tumor, which causes an outward flow that hinders the diffusion and retention of the NPs. Fortunately, the on-demand design of silicasomes allows a solution to this hindrance: Wang et al. developed an ApoA-1 mimetic peptide (R4F)-modified, indocyanine green (ICG)-loaded silicasome [31]. In this study (summarized in Figure 3), the R4F peptide, which interacts with the SR-B1 receptor present in peripheral macrophages, was incorporated into the lipid bilayer of the silicasome. This increased the uptake of silicasomes and exploited the well-known tumoritropic capacity of the macrophages and other phagocytic cells [32], enabling the nanocarrier to reach the tumor site and promoting deep tumor infiltration and distribution.

A different approach to ameliorate the reaching of the tumor cells consists of an improvement in the endothelial transcytosis. To test this alternative, Liu et al. [33] presented an irinotecan-loaded silicasome, which presented iRGD cyclic peptide on the surface, to treat pancreatic ductal adenocarcinoma in a mouse model. The results show an increased accumulation of NPs, demonstrating the increased efficacy of the coadministration of iRGD and silicasomes. A similar platform was used to deliver a combination of paclitaxel and gemcitabine and substantially outperformed a free gemcitabine + Abraxane (albumin-paclitaxel conjugate) therapy. The benefits of iRGD peptide coadministration with different drug-loaded NPs have been previously demonstrated in cancer therapeutics [34]. They can be explained by the affinity of the RGD motif for the αv integrins, specifically expressed in blood vessels undergoing angiogenesis processes, which makes it a suitable target for the constantly developing tumoral vasculature.

A risk typically associated with chemotherapy is multidrug resistance (MDR), which remains an important hindrance. This obstacle could be overcome with the development of a variety of new chemical structures that exert their therapeutic effect by different pathways to the traditional ones. In this context, natural products offer an excellent alternative due to their vast chemical diversity, enabling the discovery of novel mechanisms that allow new therapeutic strategies [35], which, in combination with nanoplatforms like silicasomes, overcome current challenges [36]. In line with this proposal, Ishaniya et al. [37] developed nanorod-shaped silicasomes to transport natural alternatives to conventional chemotherapeutic agents, with antitumoral effects. In this study, they used piperlongumine, a natural alkaloid amide isolated from long pepper that presents cytotoxic, genotoxic, antiangiogenic, and antimetastatic properties, with an adequate safety profile [38]. Although this compound presents interesting properties in cancer therapy, its low water solubility can benefit the transport within an amphiphilic nanovehicle like silicasomes. Piperlongumine was loaded in the nanosystem and administered to an in vitro MCF-7 human breast carcinoma model. The nanorod morphology has been reported to increase the cellular permeability in comparison with spherical NPs [39], contributing to the delivery effect. Results showed that lipid-coated NPs produced greater levels of apoptosis than uncoated mesoporous silica nanoparticles.

## 3. Silicasomes for Cancer Immunotherapy

Cancer immune surveillance is a physiological process in which the immune system recognizes and eliminates tumor cells. Nonetheless, malignant cells can circumvent immune recognition through a variety of mechanisms collectively known as immune escape [40]. Fortunately, over the past two decades, the field of oncology has been transformed by immunotherapy, a therapeutic approach designed to enhance the immune system’s capacity to detect and destroy cancer cells [41,42]. Despite its groundbreaking potential, immunotherapy is hindered by significant challenges, including unique toxicity profiles associated with its mechanisms of action and limited response rates [43,44]. To address these challenges, nanotechnology has emerged as a powerful strategy to boost the specificity, efficacy, and safety of cancer immunotherapy [45,46,47,48]. Among the various nanotechnological innovations, silica [49] and silica-based materials like silicasomes have drawn attention for their versatility, biocompatibility, and functional adaptability, being able to boost the activity of immunostimulatory agents in a safe and effective manner.

As previously stated, one major barrier to therapeutic success is the suboptimal pharmacokinetics and limited tumor penetration of therapeutic agents, affecting not only chemotherapeutics but also immunotherapeutics [50]. This issue is particularly prominent in immune “cold” tumors such as pancreatic ductal adenocarcinoma, which is characterized by a dense, hypovascular stroma that impedes drug delivery, restricts immune cell infiltration, and fosters an immunosuppressive tumor microenvironment, thus acting as both a physical and immunosuppressive barrier [51,52,53]. To overcome this, Luo et al. pioneered a silicasome-based nanoplatform for the delivery of Nintedanib, a triple protein tyrosine kinase (PTK) inhibitor [50]. Notably, the nanosystem enhanced drug accumulation at the tumor site, prolonged drug circulation time, and demonstrated superior therapeutic efficacy compared to oral administration. This angiokinase inhibitor potently disrupts intracellular signaling pathways mediated by members of the vascular endothelial growth factor receptor (VEGFR), platelet-derived growth factor receptor (PDGFR), and fibroblast growth factor receptor (FGFR) families, which are key regulators of tumor angiogenesis and vascular integrity [54,55]. Consequently, the delivery of encapsulated Nintedanib exhibited significant effects on vascular pruning and normalization, accompanied by a robust antitumor immune response reflected by tumor shrinkage, enhanced cytotoxic T-cell infiltration, and reduced regulatory T-cell (Treg) presence.

In addition to improving monotherapy, silicasomes offer a further advantage over conventional immunotherapy as they facilitate the simultaneous delivery of multiple immunomodulatory agents, enabling combination immunotherapy [56]. As an example, Lu et al. demonstrated a co-delivery nano approach for accomplishing robust anti-PDAC immunity through the induction of immunogenic cell death (ICD) and by interfering in the immunosuppressive indoleamine 2,3-dioxy-genase (IDO) pathway [57]. ICD is a drug-induced apoptotic process that is accompanied by the expression of calreticulin on the surfaces of dying tumor cells and the release of high-mobility group box 1 (HMGB-1) and ATP. These damage-associated molecular patterns (DAMPs) stimulate dendritic cells, fostering a pro-inflammatory environment that triggers a potent T-cell-mediated antitumor response [58]. However, ICD effectiveness is significantly reduced in the case of cold tumors due to their inherent immunosuppressive tumor microenvironment (TME). As previously mentioned, PDAC is an example of a cold tumor for which Lu et al. designed a contemporaneous delivery strategy allowed by the conjugation of Indoximod (an IDO inhibitor) to a phospholipid incorporated into a lipid bilayer encapsulating mesoporous silica NPs loaded with Oxiplatin (an ICD-inducing agent). This approach significantly enhanced the cytotoxic T-cell response and innate immunity, along with the disappearance of Tregs, resulting in improved survival outcomes without notable side effects.

When cancer cells evade the immune system, its ability for tumor eradication and function repair becomes compromised [59]. Cancer immunotherapies aim to reactivate antitumor immune responses and counteract the mechanisms of immune escape by manipulating the immune system [43]. Consequently, recent advances in chimeric antigen receptor (CAR)-T cell therapy, cancer vaccines, and immune checkpoint blockade (ICB) have become foundational approaches in cancer treatment. CARs are engineered receptors that specifically redirect T cells to recognize and destroy cells expressing a target antigen [60]. CAR-T cell therapy has revolutionized cancer treatment, especially for hematological malignancies, but there are major limitations that still must be addressed, mainly including limited efficacy in solid tumors and antigen escape, but also associated toxicities, poor CAR-T cell expansion and persistence, the immunosuppressive microenvironment, and poor trafficking and tumor infiltration [60,61,62,63]. While the application of silicasomes in CAR-T therapy is nascent [64,65,66], they have substantial potential for addressing its production needs and improving its therapeutic efficacy through combinatory strategies. For instance, Cheung et al. engineered a system that mimics natural antigen-presenting cells using a fluid lipid bilayer embedded with T-cell receptor stimulation and costimulation cues (αCD3 and αCD28 or peptide-loaded MHC and αCD28) supported by mesoporous silica microrods containing IL-2. This approach achieved faster and more substantial T-cell expansion compared to commercial expansion beads (Dynabeads) without compromising functionality in a disseminated lymphoma model [66].

Cancer cells not only evade immune recognition by downregulating or deleting the surface molecules required for antigen recognition but can also often hijack the immunosuppression system, including immune checkpoints like the PD-1/PD-L1 axis, to suppress T-cell activity at the tumor site [67]. One major limitation of therapy with immune checkpoint blocking antibodies is its ineffectiveness in certain cancers, which is linked to the “hot” or “cold” immune status of the TME, referring to the presence or absence of cytotoxic T lymphocytes, respectively, in addition to the number of immune escape mechanisms leading to resistance over time [68,69]. Additionally, the inherent relatively large molecular weight of immunoglobulins can limit their biodistribution to tumor sites, and adverse events related to antibody therapies pose significant challenges. Small-molecule immune checkpoint inhibitors (ICIs) offer a promising alternative, and advancing their delivery with nanotechnology can be game-changing to overcome systemic or off-target toxicities while improving tumor-site biodistribution and pharmacokinetics [68,70,71,72]. Allen et al. advanced a silicasome-based carrier to interfere in PD-L1 expression through glycogen synthase kinase 3 (GSK3) inhibitor (AZD1080) delivery [68]. The silicasome–AZD1080 formulation outperformed systemic administration of free AZD1080 in terms of intratumoral drug concentrations, and it was able to reduce tumor growth with comparable efficacy to anti-PD-1 in two colorectal models, pancreatic and lung cancer, whereas the free drug had no significant effect on tumor growth inhibition. In addition, the induced response was accompanied by enhanced tumor-cell killing mediated by cytotoxic T cells showing decreased PD-1 expression.

Although CAR-T cells can be effective for cancers having recognizable tumor-specific surface antigens, cancer vaccines can also stimulate immune responses against the broader set of intracellular antigens. Moreover, unlike immune checkpoint blockade (ICB) therapies that are effective primarily in subsets of “hot” tumors, cancer vaccines have the potential to prime new tumor-reactive T cells, potentially converting “cold” tumors into “hot” ones [73]. Nanovaccines are particularly advantageous for facilitating targeted delivery of antigens to critical immune organs and cell types, thereby governing the immune response and generating long-term protective immunity [59,74,75,76]. Additionally, cell-membrane-coated NPs offer a breakthrough as the resulting biomimetic nanoformulation is conferred with properties associated with the source cell, including improved biocompatibility, immune evasion, and superior targeting capacity [77,78,79]. Nguyen et al. developed a sophisticated nanoplatform using porous silica NPs coated with antigen-presenting cell-derived membranes to selectively target type 1 conventional dendritic cells (cDC1) to evoke robust stimulator of interferon genes (STING) pathway activation and promote potent antigen cross-presentation [59]. Specifically, the cDC1 subset exhibits exceptional capabilities in cross-presenting antigens and activating cytotoxic T lymphocytes. To leverage this, they developed the Si9GM nanovaccine, which consists of bone-marrow-derived dendritic cell (DC) membranes conjugated with αCLEC9A (cDC1-specific antibody) and antigen peptides (OVA257-264 tumor peptides), engulfing large-pore dendrimer-like silica NPs featuring hierarchical center-radial pores designed to deliver different sizes of αCLEC9A-OVA257-264 conjugate and STING agonist (2′3′-cGAMP). The αCLEC9A-conjugated DC membranes drive the nanovaccine directly to cDC1s, while the αCLEC9A-OVA257-264 conjugates effectively circumvent lysosomal degradation and prevent premature antigen release, enabling efficient antigen cross-presentation. Furthermore, the STING agonist 2′3′-cGAMP triggers the production of type I interferons (IFNs) as well as other proinflammatory cytokines, thereby facilitating robust CD8+ T-cell-specific antigen cross-presentation and an effective proinflammatory immune response in the TME [80]. Remarkably, the Si9GM vaccination demonstrated synergistic effects when combined with αPD-1 ICB, leading to substantial inhibition of tumor growth and metastasis in a melanoma model without notable side effects. Precisely, consistent with the induction of a proinflammatory immune response in the TME, this strategy effectively regulated M1/M2 macrophage polarization, reduced Tregs, and induced long-term immunological memory against tumor cells.

**Figure 4:**
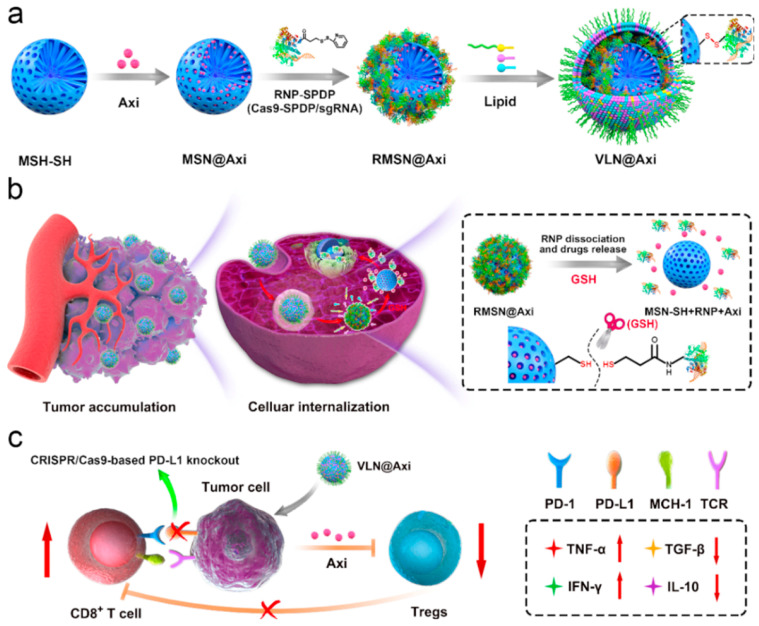
Schematic illustration of the synthesis of VLN@Axi (**a**) and delivery process (**b**) after intravenous injection. (**c**) Schematic illustration of VLN@Axi to revert the exhaustion of CD8+ T cells and suppress Tregs for efficient cancer immunotherapy. Reprinted with permission from reference [87]. Copyright © 2020 Elsevier.

Despite significant advances in immunotherapy across a broad range of cancers having been carried out, only a small subset of patients achieve durable responses and long-term survival from these therapies [81]. This limitation arises because targeting a single molecular pathway often proves insufficient for treating malignant tumors, given that multiple antitumor immunity pathways are simultaneously activated. For instance, following ICB therapy, acquired resistance can occur due to compensatory effects, such as the upregulation of alternative immune checkpoints, epigenetic modifications, disruptions in gut microbiota, or immunosuppressive elements within the TME [82]. The generation of a robust anti-tumor immune response is a complex and not entirely understood process, and cancer cells have been found to have intrinsic mechanisms bypassing every possible step along this process to evade immune detection [83,84]. Thus, in order to enhance the efficacy of cancer immunotherapy and address resistance, combination therapy strategies are increasingly being explored [82,84,85,86]. Currently, ICB is the most widely used cancer immunotherapy in clinal combination, and the key strategy behind combination therapy has been to turn immunically “cold” tumors into “hot” ones through various approaches [40,85]. For instance, several combinations of CRISPR/Cas9 systems and small-molecule drugs have shown promise in cancer treatment. However, the use of viral vectors for delivery poses challenges due to their immunogenicity and scant loading capacity. In response, Liu et al. presented a versatile nanoplatform capable of co-delivering a CRISPR/Cas9 system alongside small-molecule drugs for effective cancer therapy (Figure 4) [87]. This system used surface-thiolated mesoporous silica NPs to load small-molecule drugs into their pores, which were then sealed by conjugating the ribonucleoprotein (RNP) via disulfide bonds to respond to the reductive microenvironment typical of tumor cells. Additionally, the NPs were coated with a lipid layer to enhance circulation in the bloodstream and protect the RNP from enzymatic degradation. For validation, Axitinib, a small-molecule inhibitor of angiogenic tyrosine kinase, and a CRISPR/Cas9 system with a sgRNA targeting PD-L1 encoding gene were employed as an example of drug synergy. The resulting nanoplatform, termed VLN@Axi, successfully achieved CRISPR/Cas9-based PD-L1 knockout in cancer cells, effectively disrupting the PD-1/PD-L1 pathway and reinvigorating the exhausted T cells to suppress the tumor. Furthermore, the targeted delivery of Axitinib, which inhibits several isoforms of VEGFR, led to a reduction in the Treg population, which unleashed T-cell-mediated antitumor immunity and enhanced melanoma tumor growth inhibition. This highlights the great potential of silicasome-based systems as universal platforms for the development of advanced combination therapies against malignant tumors, paving the way for more effective and personalized cancer treatment.

Silicasomes represent a groundbreaking advancement in nanotechnology for cancer immunotherapy. By leveraging their unique properties for precise, efficient, and multifaceted therapeutic delivery, these NPs address critical limitations of conventional approaches. The most representative nanoplatforms described in this section (Table 1) underscore the diverse applications and transformative potential of silicasomes, positioning them as a cornerstone of next-generation cancer immunotherapy.

## 4. Silicasome-Based Combination Therapy

The remarkable versatility of silicasome nanosystems makes them ideal vehicles for anti-cancer combination therapy. This strategy employs multiple anti-tumoral agents within a single treatment in order to synergistically target key pathways. Research has shown that this approach not only enhances the treatment’s effectiveness but also mitigates tumor resistance. Additionally, the synergistic effect enables a greater therapeutic benefit from lower doses of each agent, which is essential for minimizing off-target toxicity and reducing adverse effects associated with treatment [88].

### 4.1. Combined Chemotherapy and Immunotherapy

**Figure 5:**
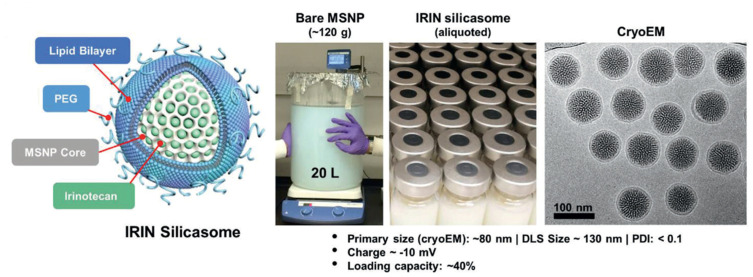
Schematic representation of structure, large-batch synthesis, and characterization via CryoEM of IRIN silicasome developed by Liu et al. Reprinted with permission from reference [90]. Distributed under the terms of the Creative Commons Attribution-Noncommercial-Noderivatives 4.0 International license. Copyright © 2021Wiley-VCH GmbH.

An example of a silicasome nanosystem that combines chemotherapy and immunotherapy for the treatment of PDAC is the irinotecan (IRIN) silicasome developed by Liu et al. [89]. IRIN functions as an ICD drug that has shown a very potent cytotoxic response and significant antitumor effectiveness. Nevertheless, its use is associated with serious side effects for the patient, which has led to the development of different strategies to deal with its adverse reactions. As an example, FOLFIRINOX is a four-drug treatment (comprising irinotecan, 5-fluorouracil, oxaliplatin, and leucovorin) restricted to patients in a relatively good health situation able to tolerate the consequences of the treatment. Onivyde is another FDA-approved example of an IRIN administration alternative, which consists of an IRIN liposomal formulation that provides a better survival rate but suffers from drug leakage. To overcome all these inconveniences, Liu et al. have designed IRIN-encapsulated silicasomes that have been tested in immunocompetent mice, showing better therapeutic effect than the other IRIN formulations [89]. However, as a consequence of the treatment, potential immunosuppressive effects resulting from the IRIN-induced PD-L1 expression could appear. For this reason, in a subsequent workthe silicasome carrier was combined with an intraperitoneal injection of anti-PD-1 antibodies (Figure 5). In a survival experiment with a Kras-induced pancreatic cancer orthotopic model, it was observed that IRIN silicasome + anti-PD-1 worked significantly better than monotherapy, as the antibodies enhanced T-cell cytotoxicity and ICD response. Furthermore, the IRIN silicasome + anti-PD-1 combination therapy also outperformed Onivyde + anti-PD-1, increasing the median survival time from 29 to 43 days [90]. This research concluded that, while Onivyde liposomes achieved a concentration of only 0.328 ± 0.128 μg IRIN/g tumor 48 h post-treatment, the silicasome system reached a much higher concentration of 9.545 ± 5.103 μg IRIN/g tumor [91].

In a subsequent study, this silicasome-based IRIN + anti-PD-1 treatment was adapted for advanced colorectal cancer, which called for the addition of a radiotherapeutic stimulus [91]. It has been established that radiation therapy can damage tumor vascular endothelial cells and enhance the permeability of NPs to the tumor [92]. Furthermore, radiation can amplify IRIN-mediated ICD response through the cGAS/STING-dependent type-I interferon (IFN) pathway [91,93]. Wang et al. demonstrated that combining IRIN silicasomes with X-ray irradiation significantly improved their ability to produce reactive oxygen species and induce DNA damage, causing an increase in type I IFN production. This phenomenon not only improved tumor cell antigen presentation but also resulted in an upregulation of PD-L1 that was effectively blocked by the anti-PD-1 antibodies. As a result, the treatment with IRIN silicasome + radiation led to an increased proportion of CD4+ and CD8+ T cells recruited to the tumors in comparison with monotherapy, and this effectiveness was further enhanced with the addition of anti-PD-1 treatment [91]. Thus, this research sets a promising example for the combination of chemotherapy, immunotherapy, and radiotherapy through the use of silicasomes. Luo et al. developed another iteration of the chemo-immunotherapeutic IRIN silicasome through the incorporation of 3M-052, a TLR7/8 agonist, into the lipid coating. In vivo experimentation demonstrated a synergistic anti-PDAC immune response due to 3M-052′s ability to boost the antigen-presenting function of dendritic cells. The co-delivery of IRIN and 3M-052 achieved significantly greater tumor shrinkage compared to monotherapy and, more notably, it increased dendritic cell activation at both the tumor site and regional lymph nodes [94].

### 4.2. Combined Chemotherapy and Radiotherapy

**Figure 6:**
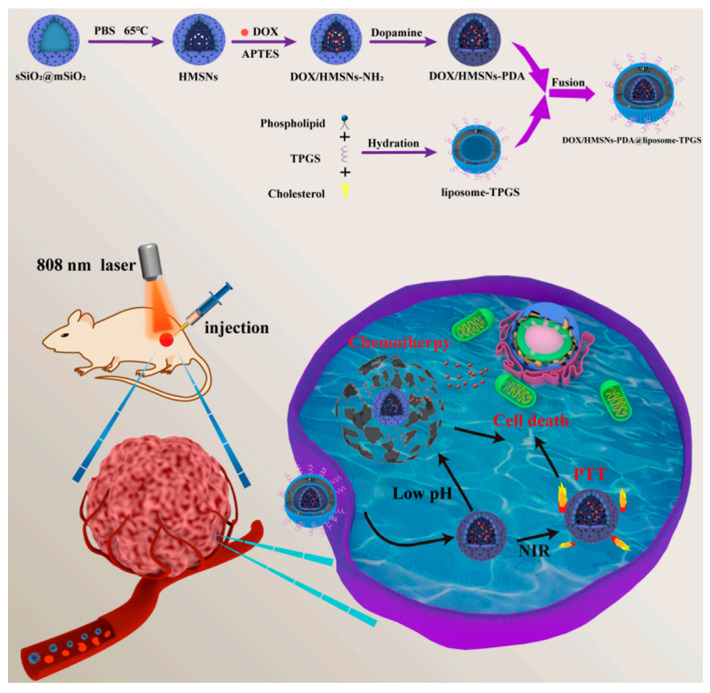
Schematic representation of the construction of the DOX + polydopamine silicasome developed by Fan et al. for pH/NIR-responsive drug release and chemo-photothermal therapy. Reprinted with permission from reference [95]. Distributed under the terms of the Creative Commons Attribution-Noncommercial-Noderivatives 4.0 International license. Copyright © 2023 MDPI.

Although immunotherapy is the most typical pairing for chemotherapy, silicasomes allow the synthesis of a plethora of different combinations. For instance, Fan et al. developed a system that integrates the transport of DOX (DOX) with photothermal therapy. In this approach, DOX is loaded into hollow mesoporous silica, which is initially coated with a polydopamine (PDA) film and subsequently covered with a D-α-tocopheryl PEG-1000 succinate (TPGS)-modified lipid membrane. PDA serves as an effective light-to-heat converter when subjected to near-infrared (NIR) irradiation, giving the silicasome a photothermal conversion efficiency of 16.7% and enabling local hyperthermia treatment in tumor regions (Figure 6) [95]. The TGS-modified lipid layer has been shown to improve in vitro cytotoxicity against cancer cells and reverse MDR [96]. In this case, it enhanced blood circulation time and biocompatibility. In comparison, bare MSNPs caused complete hemolysis when their concentration exceeded 400 µg/mL, while the silicasomes did not exhibit visible hemolysis even at concentrations as high as 800 µg/mL. Additionally, the silicasome co-loading system demonstrated a synergistic effect, as DOX uptake was 2.60 times higher in tumor samples treated with NIR irradiation due to increased cell metabolism and enhanced cell membrane permeability [95].

Another instance where silicasomes have been paired with radiation to combat oncological conditions is through photodynamic therapy (PDT). This approach utilizes specific photosensitizer (PS) compounds that produce highly reactive singlet oxygen (^1^O_2_) in response to light excitation. This results in ROS-mediated toxicity, which can effectively target tumors in three key ways: by inducing apoptosis and/or necrosis in tumor cells, by damaging the tumor’s vasculature, and by activating an immune response [97]. As a notable example, Teng et al. incorporated the PS protoporphyrin IX into folate-functionalized silicasomes, achieving very encouraging results both in vitro and in vivo [98]. For their part, Ma et al. devised a more sophisticated silicasome system to combine PDT with chemotherapy. With this purpose, DOX was loaded into a mesoporous silica core that was firstly coated with a calcium phosphate interlayer to achieve a pH-triggered drug release, and then encapsulated with a PEGylated liposome containing the second-generation PS zinc (II) phthalocyanine (ZnPc). In this case, the calcium interlayer not only allowed for the controlled release of DOX but also promoted intracellular uptake of the nanoparticles and induced lysosomal disruption due to an osmotic pressure imbalance. Additionally, the silicasome was proved to have an excellent antitumoral effect through synergetic chemotherapy-PDT in vitro [99].

### 4.3. Use of Biomolecules for Combined Therapy

**Figure 7:**
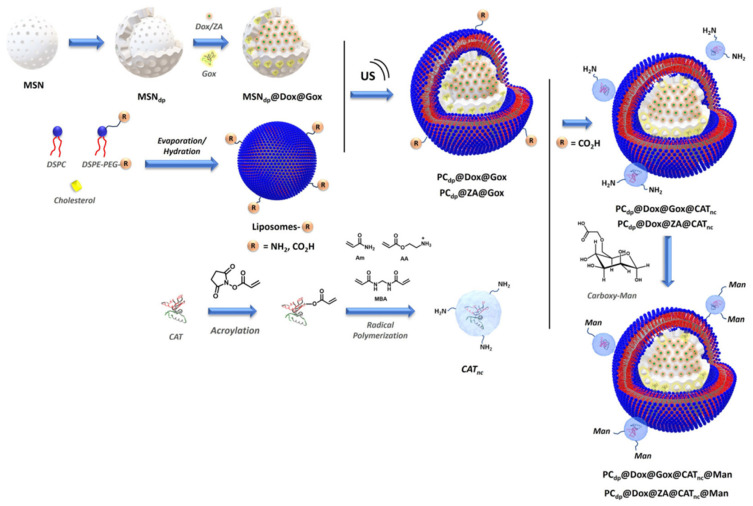
Schematic representation of the synthetic pathway to obtain the double porous silicasomes modified with polymeric nanocapsules [100]. Copyright © 2024 The Royal Society of Chemistry.

In addition to immunotherapy and radiotherapy, the use of biomolecules like enzymes or nucleic acids in combination with chemotherapy has been described in the literature. Parra-Nieto et al. designed a smart approach that consisted of the encapsulation of DOX within the silicasomes and the incorporation of polymeric nanocapsules that contained glucose oxidase enzyme on the surface of the silicasomes. This strategy permitted the delivery of a selected chemotherapeutic agent in combination with the action of the enzyme that provoked glucose starvation and oxidative damage [25]. This platform was exceeded in a subsequent work in which a double-pore mesoporous silica core was used. This double porosity was used to house both DOX and glucose oxidase enzyme while, on the outer surface of silicasomes, polymeric nanocapsules were loaded with catalase enzyme. This nanodevice was proven to successfully release DOX and contribute to maintaining the oxygen level in the tissue through an enzymatic cascade reaction (Figure 7) [100].

In addition to enzymes, nucleic acids have also been used in combination with chemotherapy: Xue et al. combined the transport of DOX with that of miR-375 to overcome P-glycoprotein (P-gp)-mediated MDR in hepatocellular carcinoma [101]. The microRNA (miRNA) miR-375 bound to the 3′ untranslated region of the astrocyte elevated gene-1 (AEG-1), blocking its translation through RNA interference (RNAi). This action inhibited AEG-1′s role in promoting P-gp production [102]. Due to the negative charge of miR-375, it could not be co-loaded with DOX into the anionic hollow MSNPs and, instead, was integrated into a cationic lipid layer that coated the nanoplatform. It was observed that this lipid layer stabilized the silicasome and decreased the premature release of DOX, reducing hepatic and cardiac toxicity in mouse models. Furthermore, the presence of miR-375 effectively reduced the percentage of DOX pumped out of the HEpG2/ADR cells by P-gp (from 80% in the case of the free drug to 28%) and significantly increased tumor cytotoxicity by contributing to mitochondria-primed apoptosis. These results highlight the synergistic co-efficiency between DOX and miR-375, facilitated by silicasome transportation [101].

**Figure 8:**
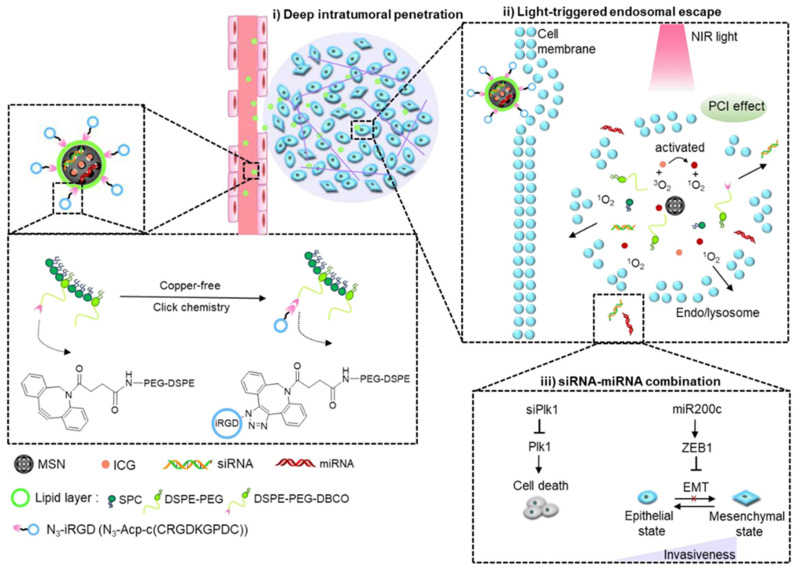
Schematic Illustration of light-triggered RNA delivery by tumor-penetrating iRGD + indocyanine green + siPlk1/miR-200c silicasome developed by Wang et al. Reprinted with permission from reference [103]. Copyright © 2020 American Chemical Society.

Despite the vast presence of chemotherapy in anticancer approaches, the combination of other therapeutic agents has been described in the literature. Wang et al. proposed the transport and delivery of siPlk1 small interfering RNA (siRNA) and miR-200c microRNA (miRNA) within silicasomes to transport genetic material (Figure 8) [103]. The siPlk1 silences the expression of Polo-like kinase 1 (Plk1), which inhibits cell proliferation and induces apoptosis, while miR-200c suppresses metastasis by hindering epithelial-to-mesenchymal transition (EMT) [104,105]. Although this siRNA/miRNA combination shows significant promise for oncology treatment, it is greatly limited by the availability of safe and efficient systemic delivery systems with sufficient tumor-penetrating and endosomal-escaping capabilities. As a means to solve this, Wang et al. complemented their delivery with the use of the indocyanine green (ICG) photosensitizer, to facilitate light-triggered endosomal escape, and the cyclic tumor-penetrating peptide iRGD (CRGDK/RGPD/EC) [103]. This iRGD peptide functions by interacting with αν integrins and the neuropilin-1 receptor to induce a three-step endocytic transport pathway and has shown excellent results in enhancing deep tumor penetration of silicasomes through PDAC desmoplastic stroma [33]. Thus, to create this silicasome, a mixture of siPlk1 and miR-200c was adsorbed on the surface of the mesoporous silica core loaded with ICG, which was then coated with an iRGD-conjugated lipid layer. The results indicated that the iRGD lipid coating improved drug delivery to breast cancer cells, while the ICG photodynamic treatment facilitated siRNA/miRNA escape from endosomes, significantly boosting their antitumor response. The complete nanosystem demonstrated higher tumor growth inhibition compared with monotherapies and effectively reduced lung metastasis from 100% to 40% in a highly metastatic orthotopic MDA-MB-231 breast tumor model [103].

### 4.4. Silicasomes as a Theranostic Tool

As theranostics have become increasingly important in the field of oncology, researchers have developed various silicasome treatments that involve the use of imaging techniques. Silicasomes have been identified as very sophisticated active ultrasound platforms that, unlike traditional ultrasound agents such as stabilized perfluorocarbon microbubbles or stabilized liquid droplets, can accumulate in tumors and internalize into cancer cells. This unique capability allows silicasomes to serve as highly effective enhancers for high-intensity focused ultrasound (HIFU) ablation [106]. Conventional HIFU therapy uses high acoustic ultrasound doses in order to significantly increase tissue temperature (>50 °C) and lead to effective tumor necrosis. However, this method presents some drawbacks: delivering the necessary acoustic doses for thermal ablation can be challenging, and there is a risk of toxicity to healthy tissue due to non-specific hyperthermia over a relatively large area [107]. In contrast, silicasomes enable localized mechanical HIFU ablation through the generation of cavitation bubbles, which can grow up to 100–200 μm before collapsing and causing severe cellular damage [106,108]. Building on the knowledge that under reduced acoustic pressures, hydrophobic MSNPs can create this cavitation effect, Yildrim et al. enhanced this system by coating it with a folic acid-conjugated lipid layer. This modification allows targeted delivery of the nanosystem without altering the MSNP surface, which serves as the nucleation site for bubble formation. Using a human breast adenocarcinoma in vitro model, it was demonstrated that these silicasomes effectively accumulated in tumors and mechanically ablated the target cancer cells with a minimal temperature increase of 3.5 °C, which significantly reduced damage to the surrounding tissue and vasculature [106].

In a follow-up study, it was determined that the cavitation ability of the silicasomes was highly dependent on the behavior of the lipid phases. Specifically, it was observed that bubbles were only generated when the lipids were in a gel phase below their melting temperature, and not when they were in a liquid expanded phase. This phenomenon was employed by Blum et al. to design a silicasome theranostic system that could “switch off” its ultrasound capabilities in response to temperature changes. By carefully selecting their lipid compositions, they were able to create silicasomes that silenced their acoustic contrast at temperatures ranging from 20 °C to 55 °C, which has intriguing applications not only as a temperature sensor but also for self-regulated thermometry during HIFU application [109].

Another powerful tool that can be used to adapt silicasomes to a theranostic approach is the incorporation of superparamagnetic iron oxide nanoparticles (SPIONs) into the core of the nanodevice. This allows them to garner their great potential as contrast agents in magnetic resonance imaging (MRI) and cell hyperthermia inducers [110]. Following this line of action, Patil-Sen et al. developed a Fe_3_O_4_ SPION-core silicasome co-loaded with DOX. The results indicated that the double coating retained the ultrasmall size and superparamagnetic characteristics of SPIONs and, even more, increased their colloidal stability. In regard to their diagnostic ability, the silicasome system showed a significant shortening of T2 relaxation time, from 1316 ms in the free SPIONs to 46 ms, which is an auspicious result for the improvement of MRI image quality. Additionally, in vitro evaluations showed the suitability of these nanoparticles for hyperthermia drug delivery applications and their increased biocompatibility towards non-malignant cells [111]. Similarly, Liu et al. devised a Fe_3_O_4_ SPION-core silicasome loaded with DOX in the mesoporous silica and coated with a ZnPc-methotrexate conjugated liposome to combine chemo-PDT therapy with multimodal imaging. This complex nanosystem integrates fluorescent, MRI, and photoacoustic imaging to achieve accurate diagnosis and monitoring of oncological pathologies. Moreover, through the combination of magnet targeting with methotrexate-mediated endocytosis, it is able to successfully deliver the synergistic therapeutic effects of both DOX chemotherapy and ZnPc PDT to the tumor while reducing off-target toxicities [112].

Lastly, an excellent example of combinatorial theranostic silicasome has been conceived by Palanikumar et al. to merge multimodal diagnostic imaging with PDT and photothermal therapy. With this purpose, a solid core composed of sodium yttrium fluoride upconversion nanoparticles doped with lanthanides and bismuth selenide (NaYF_4_:Yb/Er/Gd, Bi_2_Se_3_) was coated with a chlorin e6-loaded silica layer and encapsulated within a PEGylated liposome. In this system, the upconversion nanoparticles NaYF4:Yb/Er convert NIR into visible light, triggering excitation of PS chlorin e6 and its subsequent ROS production. Simultaneously, Bi_2_Se_3_ converts that same NIR light to heat, and Gd enables MRI to coordinate this complex therapeutic delivery. The synergistic nature of this treatment can be explained by the ROS-mediated inactivation of heat shock proteins and the hyperthermia-induced O_2_ accumulation in tumor tissue [113].

## 5. Conclusions

Nanotechnology has provided new strategies to deliver therapeutic agents to solid tumors in a more controlled and selective manner. The discovery of the EPR effect triggered the race to engineer smart nanocarriers capable of delivering chemotherapy to solid tumors exploiting the characteristic aberrant vasculature of cancerous tissue. During the last decades, a myriad of different nanodevices—organic, inorganic, or hybrid—have been reported, each of them with its respective strengths and weaknesses. The common advantage that these nanodelivered therapies present over traditional strategies is a decrease in side effects owing to their accumulation in the tumor microenvironment because of the presence of a leaky vasculature. Among them, silicasomes constitute powerful nanoplatforms due to their hybrid nature, which combines the excellent cargo capacity and controlled-release kinetics of silica NPs with the high colloidal stability, biocompatibility, capacity to house hydrophobic drugs, and tunability of liposomes. Herein, some of the most representative examples of their application in antitumoral therapy have been presented to provide an insight into the potential of this nanoplatform.

## Figures and Tables

**Table 1:** Selected examples of silicasome-based nanoplatforms developed for cancer immunotherapy.

Mechanism of Action	Target Cell	Payload	Additional Features	Tumor Model	Ref
PTK inhibition	Stroma and tumor cells	Nintedanib	-	PDAC	[50]
ICD + IDO inhibition	Tumor cells	Indoximod + Oxiplatin	-	PDAC	[57]
T-cell expansion	T cells	IL-2	αCD3 and αCD28 or peptide-loaded MHC and αCD28 anchored in the fluid lipid bilayer	B-cell lymphoma	[66]
PD-L1 inhibition	Tumor cells	AZD1080	-	Colorectal, pancreatic, and lung cancer	[68]
Cancer vaccination	cDC1 cells	αCLEC9A-OVA257-264 conjugate + 2′3′-cGAMP	NPs with hierarchical center-radial pores + DC cell membranes anchored with αCLEC9A-OVA257-264	Melanoma	[59]
CRISPR/Cas9-based PD-L1 knockout + tyrosine kinase inhibition	Tumor cells	Axitinib + Cas9-SPDP/sgPD-L1	-	Melanoma	[87]

PDAC: pancreatic ductal adenocarcinoma; ICD: immunogenic cell death; IDO: indoleamine 2,3-dioxy-genase; MHC: major histocompatibility complex.

## Data Availability

Not applicable.
